# Use of the ureteral access sheath during ureteroscopy: A systematic review and meta-analysis

**DOI:** 10.1371/journal.pone.0193600

**Published:** 2018-02-28

**Authors:** Jian Huang, Zhijian Zhao, Jad Khaled AlSmadi, Xiongfa Liang, Fangling Zhong, Tao Zeng, Weizhou Wu, Tuo Deng, Yongchang Lai, Luhao Liu, Guohua Zeng, Wenqi Wu

**Affiliations:** 1 Department of Urology, Minimally Invasive Surgery center, Guangzhou Institute of Urology, Guangdong Key Laboratory of Urology, the First Affiliated Hospital of Guangzhou Medical University, Guangzhou, Guangdong, China; 2 Department of organ transplantation, The Second Affiliated Hospital of Guangzhou Medical University, Guangzhou, Guangdong, China; Sun Yat-sen University, CHINA

## Abstract

The debate still rages on for the usefulness of ureteral access sheath (UAS). Therefore, a meta-analysis to discuss the effects of applying UAS during ureteroscopy was performed. The protocol for the review is available on PROSPERO (CRD42017052327). A literature search was conducted up to November, 2017 using the Web of science, PUBMED, EMBASE and Cochrane Library. The quality of articles was assessed by the Jadad scale and Newcastle Ottawa Scale (NOS). Egger’s test and the trim-and-fill method were used to evaluate publication bias. Effect sizes were calculated by pooled odds ratio (ORs) and mean differences (MDs). Sensitivity analyses and subgroup analyses were performed to explore the origin of heterogeneity. Eight trials with a total of 3099 patients and 3127 procedures were identified. Results showed no significant difference in stone-free rate (SFR) (OR = 0.83, 95% CI 0.52–1.33, P = 0.45), intraoperative complications (OR = 1.16, 95% CI 0.81–7.69, P = 0.88), operative time (MD = 4.09, 95% CI -15.08–23.26, P = 0.68) and hospitalization duration (MD = -0.13, 95% CI -0.32–0.06, P = 0.18). However, the incidence of postoperative complications was higher in UAS group (OR = 1.46, 95% CI 1.06–2.00, P = 0.02). Evidence from meta-analysis indicated that the use of UAS during ureteroscopy did not manifest advantages. However, given the intrinsic restrictions of the quality of selected articles, more randomized controlled trials (RCTs) are warranted to update the findings of this analysis.

## Introduction

Ureteral access sheath (UAS) was first introduced in 1974 to facilitate passing ureteroscope into the ureter [1]. With the progress of technology, hydrophilic coating of UAS minimized shear force and enabled smoother passage of the sheath into the ureter, hub-locking mechanism enabled the sheath and the dilator to be passed through the ureter as a whole one unit. Those modifications have increased the safety and wide use of UAS [2]. Theoretically, it provides an access to the collecting system with the ability for multiple entries and exits of the ureter, allowing for evaluating any portion of the kidney, decreasing intrarenal pressure during irrigation, and improving drainage around the scope and visibility, thus protecting the scope when performing lithotripsy and extracting stone fragments [3–6]. Nevertheless, there have been some misgivings concerning UAS use and the risk of ureteral injury [7]. Whether the UAS is an efficacious equipment remains hang in the wind and the debate rages on. Several studies on this issue had been conducted, while the results were contradictory [8–15]. Here, we performed a systematic review and meta-analysis based on current evidence to assess the effectiveness and safety of the use of ureteral access sheath during ureteroscopy, in aim to conclude an evidence-based recommendation for clinical practice.

## Materials and methods

### Search strategy

This systematic review and meta-analysis was reported according to the Preferred Reporting Items for Systematic Reviews and Meta-Analyses (PRISMA) Statement. The protocol for the review was available on PROSPERO (CRD42017052327; https://www.crd.york.ac.uk/prospero/). Literature retrieval was conducted up to November, 2017 using Web of science, PUBMED, EMBASE and Cochrane Library. The following Medical Subject Heading (MeSH) or Emtree terms combined free terms of “ureteroscopy” and “ureteral access sheath” were searched in different databases. The methodology filters about study design was derived from the Francis A. Countway Library of Medicine (http://guides.library.harvard.edu/meta-analysis). Additionally, we performed a manual search from references of included articles to retrieve other applicable studies. There were no language or publication date restriction in retrieval strategy.

### Selection criteria

All processes including literature search, selection criteria, data extraction, quality assessments, and statistical analyses were performed by two authors independently and double-checked by both. Any divergence was disposed by a senior author. Eligibility criteria for the included studies were defined base on the PICOS principles: (1) Participants (P): Patients were diagnosed or treated by ureteroscopy. (2) Interventions (I) and comparisons (C): Exploring the effect of UAS during ureteroscopy. (3) Outcomes (O): Including at least one of the following outcomes: stone-free rate (SFR), intraoperative or postoperative complications, operative time and the hospitalization time. (4) Study design (S): RCTs or comparative studies with the relative data could be used directly or indirectly. Articles met the following points were abandoned: (1) Letters, review articles, laboratory studies, case reports and animal experimental studies. (2) Absence of key information such as sample size, 95% CI, and P value or this value could not be calculated. (3) The study design without a comparative group. (4) A repetitive publication article.

### Data extraction

The relevant data of included studies were extracted with a well-designed form. Data collectors were blinded to authors or journals. The following data were extracted from each article independently: sample sizes, procedures performed, study period, country, follow-up time, article type, age, stone burden, preoperative or postoperative stent, outcomes and so on. The collecting data were gathered with the primitive form from article to insure the veracity. Only the optimal research design and the most holonomic data included when a repeated article was met. We contacted the original authors to obtain more detail information when necessary. The primary outcome was SFR. The secondary outcomes included intraoperative and postoperative complications, operative time and hospital stay.

### Quality assessments

The quality of RCTs were assessed with Jadad scale [16]. A 9-score system named Newcastle Ottawa Scale (NOS) [17,18] was used when non randomized controlled trials (non-RCTs) were met. Studies estimated with this system were considered of high-quality if achieved a score of seven or more.

### Statistical analysis

The relevant data were analyzed by Review Manager Version 5.3 software (Review Manager, Version 5.3 for Windows, The Cochrane Collaboration, 2013) and STATA 12.0 (StataCorp, College Station, TX, USA). Pooled odds ratios (ORs) and mean differences (MDs) were respectively calculated as the summary statistic for dichotomous variables and continuous variables with 95% confidence intervals (CIs). The Z test was used to analyze pooled effects, and a P-value of less than 0.05 was considered statistically significant. The standard deviation (SD) was converted by sample size and range using the theory described by Hozo et al if necessary [19]. Heterogeneity was appraised using Cochran’s Q test (reported with a x^2^-value and P-value) and I^2^ statistic [20,21]. A P-value of less than 0.1 means the presence of heterogeneity when the Q test was performed. An I^2^ value of more than 50% was considered an indication for moderate to serious heterogeneity. Reasons for statistical heterogeneity were explored with sensitivity analyses. Sensitivity analyses were conducted by exclusion of individual studies. A random-effects model was used for pooling when there was evidence of heterogeneity [22]. Otherwise, the combined estimates were shown with a fixed-effects model [23]. Subgroup analyses were performed according to patient age, stone site, study design, the control of stone size, and the bias from semirigid ureteroscopy to evaluate UAS and non-UAS groups. The Egger linear regression test [24] and the non-parametric trim-and-fill method [25] were conducted to explore the publication bias for SFR.

## Results

### Literature search

**Fig 1:**
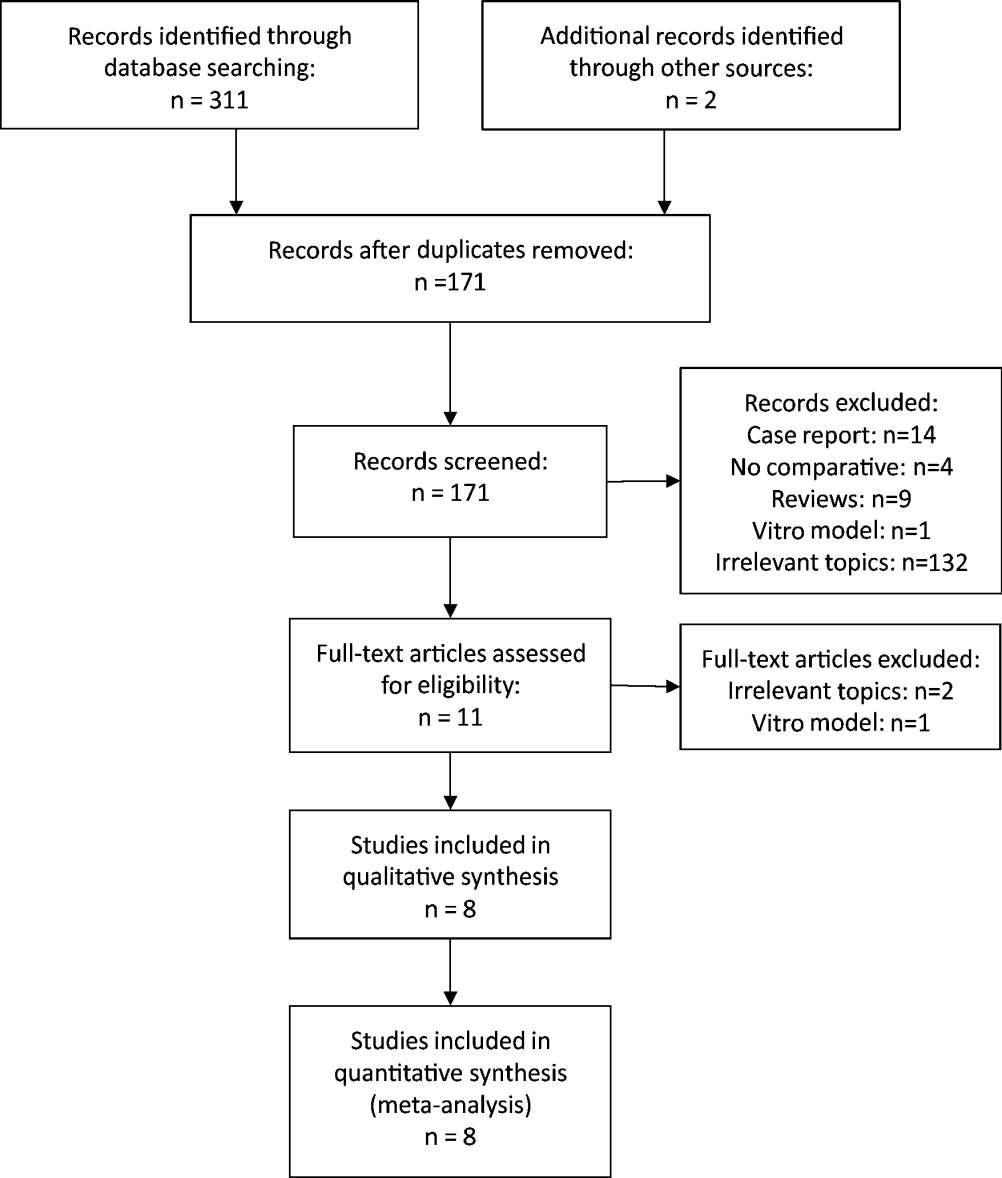
Flow diagram of studies selection process.

The whole process of literature search was presented in Fig 1. The initial search identified 311 potentially relevant studies. Additionally, two studies were available by manual search with references. Then 142 duplicates were distinguished and excluded by the “duplicates check” function of NoteExpress. After the exclusion of case reports, non-comparative studies, reviews, in-vitro model studies and irrelevant-topic studies with the first browse of title and abstract, the full-text versions of 11 papers were identified to determine eligibility. In these articles, two studies were recognized as irrelevant topics, one of them was associated with an in-vitro model. Finally, eight eligible studies [8–15] including 3099 patients, a total of 3127 procedures of which 1994 procedures for UAS group and 1133 procedures for non-UAS group, fulfilling the inclusion criteria were recognized.

### Study characteristics and quality assessment

Included trials were published between 2001 and 2016, and conducted in the United States (3), Switzerland (1), Greece (1), France (2), and England (1). The sample sizes ranged from 28 to 2239. Among the 8 included studies, there were 2 RCTs [8,11] between UAS group and non-UAS group. Two studies belong to prospective cohort studies [14,15], four studies were classified to be retrospective cohort studies [9,10,12,13]. The quality of included non-randomized studies were granted a score between 5 and 7. Both of RCTs got 2 scores on the Jadad scale (Table 1). One of the included studies was related to pediatric stone management [12] and three of them were involved in distal ureteral abnormalities [9,11,12].

**Table 1:** UAS versus non-UAS: Summary of selected studies.

Reference	Sample sizes/procedures performed, n	Study period	Country	Follow-up time	Article type	Quality score
Kourambas 2001	59/62	Oct. 1999-Jan. 2000	United States	3 month	RCT	2^b^
De Sio 2004	28	1999 to May 2003	Switzerland	NR	Retrospective cohort study	5^a^
L'esperance 2005	256	1997 to 2003	United States	2 month	Retrospective cohort study	7^a^
Pardalidis 2006	98	Jan. 2001 to Dec. 2004	Greece	1 year	RCT	2^b^
Wang 2011	96	1999 to 2009	United States	11 month (0.2-110month)	Retrospective cohort study	5^a^
Berquet 2014	280	2009 to 2012	France	1–3 month	Retrospective cohort study	7^a^
Traxer 2015	2239	Jan. 2010 to Oct. 2012	France	NR	Prospective cohort study	5^a^
Geraghty 2016	43/68	Mar. 2012 to Oct. 2014	England	2–3 month	Prospective cohort study	6^a^

RCT randomized controlled trials, NR not reported.

^a^ Using Newcastle-Ottawa Scale (score from 0 to 9).

^b^ Using Jadad scale (score from 0 to 5).

The baseline characteristics of the relevant literatures were indicated in Table 2. The adjusted variables were recorded to develop a better understanding of the comparability between groups. A very important parameter, stone burden, was no significant differences in 6 original documents [8,10,11,13–15].

**Table 2:** Characteristics of selected studies.

Reference	Group	Procedures, n	Age, years	Stone burden, mm	Preoperative stent, procedures, n (%)	Postoperative stent, procedures, n(%)	Flexible or semirigid ureteroscopy, F:S	The definition of SFR	Adjusted variable
Kourambas 2001	UAS	30	43.8 (21–85)	13.00	NR	15	25:5	NR	Stone burden, the use of flexible or semirigid ureteroscopy, fragmentation device
	Non-UAS	32		10.35		19	23:9		
De Sio 2004	UAS	12	54 (26–71) and 61 (54–68)	1.4 (1–2.5) and 0.7 (0.4–0.9)	NR	NR	Semirigid ureteroscopy	NR	NR
	Non-UAS	16	45 (18–74) and 63 (61–75)	1.6 (1.1–2.8) and 0.9 (0.4–1.2)					
L'esperance 2005	UAS	173	49	8.7	NR	77%	Flexible ureteroscopy	Completely clean	Age, gender, stone burden, stone location, postoperative stent
	Non-UAS	83	47	7.3					
Pardalidis 2006	UAS	48	48.5 (18–73)	7.1	NR	100%	Flexible ureteroscopy	Completely clean	Stone burden, Use of EHL
	Non-UAS	50		7.8					
Wang 2011	UAS	40	13.6±4.2 (4.0–20.9)	12.5 ± 9.7 (3.0–54.0)	12	38	NR	NR	Age, BMI, gender, race, urinary tract abnormalities, preoperative stent
	Non-UAS	56	12.7±4.6 (1.5–19.9)	7.6 ±4.5 (0.8–27.0)	14	37			
Berquet 2014	UAS	157	50±15.2	15.15±9.8	39 (24%)	134 (85%)	Flexible ureteroscopy	Residual stone ≦3mm	Age, stone number, stone burden, postoperative stent
	Non-UAS	123	52±17.3	13.75±8.0	62 (50%)	94 (76%)			
Traxer 2015	UAS	1494	51.2±14.98	108.3±114.4^a^	511	1352	Flexible ureteroscopy	Residual stone ≦1mm	Age, gender, BMI, renal congenital abnormality, stone burden, previous calculus treatment, solitary kidney, preoperative stent, case volume
	Non-UAS	745	50.2±14.95	99.2±100.5^a^	278	611	Flexible ureteroscopy		
Geraghty 2016	UAS	40	54 (7–84)	29.2 (20–60)	15	64 (94.1%)	Flexible ureteroscopy	Residual stone ≦2mm	Stone burden
	Non-UAS	28			11				

NR not reported, SFR stone free rate, UAS ureteral access sheath, non-UAS without an ureteral access sheath, BMI Body Mass Index, F:S Flexible ureteroscopy: Semirigid ureteroscopy, EHL electrohydraulic lithotripsy, SD standard deviation.

Values are given as mean ± SD (range).

^a^ Stone burden was calculated as the sum of all stone sizes (length × width × 0.25 × 3.14159).

### Primary outcomes

#### SFR

**Fig 2:**
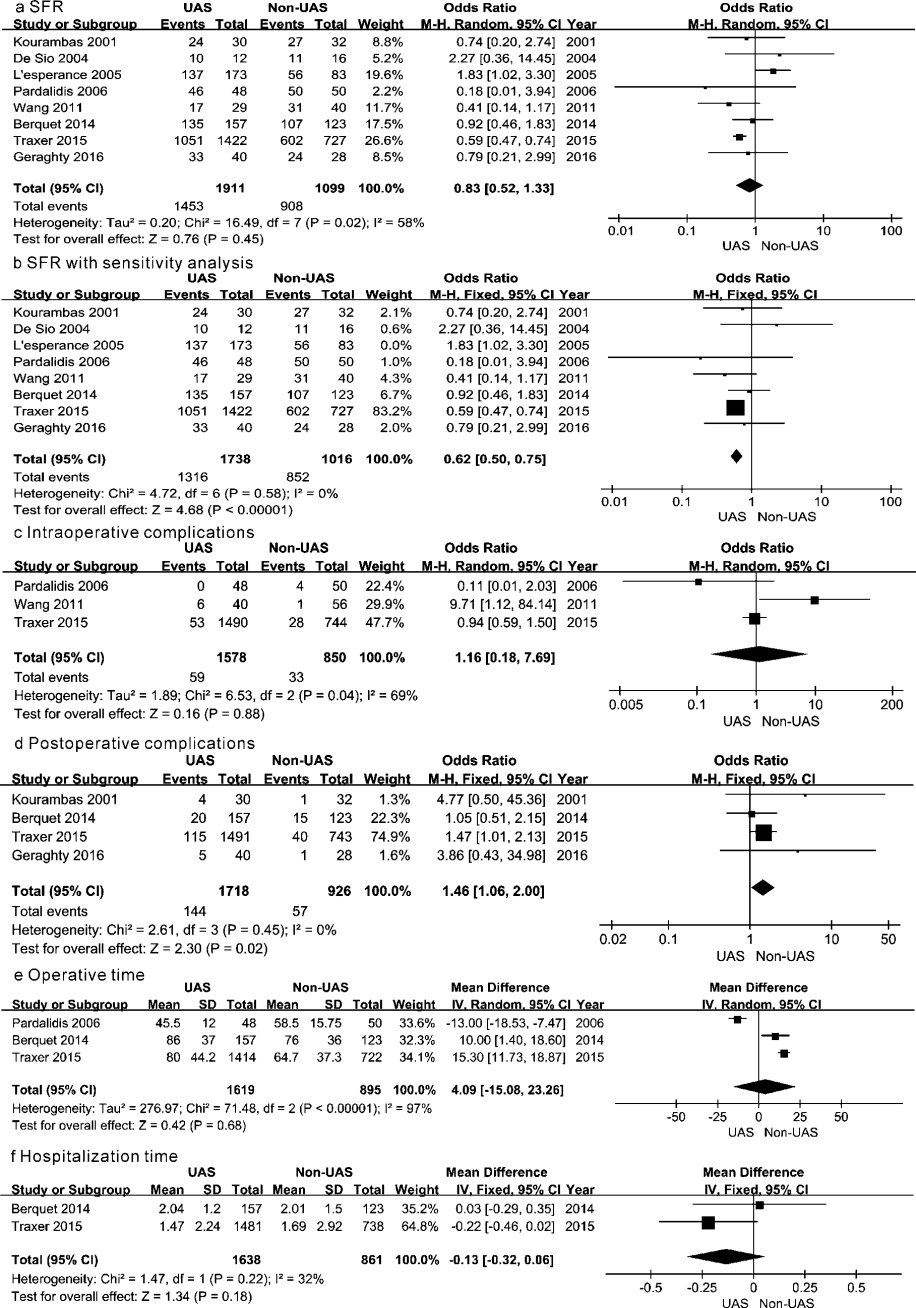
Forest plot in meta-analysis. a SFR, b SFR with sensitivity analysis, c intraoperative complications, d postoperative complications, e operative time, f hospitalization time. UAS: ureteral access sheath; non-UAS: without an ureteral access sheath.

The definition of SFR was made in five studies [10,11,13–15]. There were two studies [10,11] identified SFR as having none of the stone left. One of the article [14] recognized it as residual stone less than 1 mm, while one [15] defined it as having a residual stone fragment that is less than 2 mm, and the other one [13] described it as residual stone less than 3 mm. Computed Tomography scan and X-ray were the principal imaging examination to evaluate the residual stone. All studies have compared the difference of SFR between the UAS and non-UAS groups. The data of each articles were summarized with a random effects model and showed no significant difference between arms (OR = 0.83, 95% CI 0.52–1.33, P = 0.45, Fig 2A). This result showed moderate heterogeneity (Q = 16.49, P = 0.02, I^2^ = 58%, Fig 2A). Sensitivity analysis by removing individual studies showed clinical heterogeneity of being due to the article of L’esperance et al [10]. Combining results of the remaining 7 studies with fixed effects model demonstrated a higher SFR for the non-UAS group rather than the UAS group (OR = 0.62, 95% CI 0.50–0.75, P < 0.00001, Fig 2B) and revealed no heterogeneity between groups (Q = 4.72, P = 0.58, I^2^ = 0%, Fig 2B).

#### Subgroup analysis

Subgroup analyses were conducted on account of age, diseased region, study design, the control of stone size, and the use of semirigid ureteroscopy to further estimate the efficiency of UAS in SFR. However, only the sort of prospective cohort study [14,15] in the subgroup analysis of study designs presented the different result with the original outcome, which was favoured of non-UAS group (OR = 0.59, 95% CI 0.47–0.74, P < 0.00001; Q = 0.18, P = 0.68, I^2^ = 0%, Table 3).

**Table 3:** Subgroup analysis.

subgroup	Studies, n	Heterogeneity			OR (95% CI)	P value
		Q	P value	I^2^, %		
Age						
Adult	7	15.50	0.02	61	0.92 (0.55–1.54)	0.74
Children	1	-	-	-	0.41 (0.14–1.17)	0.10
Diseased region						
Proximal	5	13.22	0.01	70	0.90 (0.52–1.56)	0.72
Distal	3	3.04	0.22	34	0.62 (0.18–2.16)	0.45
Study design						
RCT	2	0.68	0.41	0	0.60 (0.18–1.99)	0.40
Prospective cohort study	2	0.18	0.68	0	0.59 (0.47–0.74)	<0.00001
Retrospective cohort study	4	7.05	0.07	57	1.08 (0.54–2.14)	0.84
The control of stone burden						
Control	6	13.96	0.02	64	0.86 (0.51–1.47)	0.59
Uncontrolled	2	2.49	0.11	60	0.81 (0.16–4.18)	0.80
Extract/shatter stone with flexible or semirigid Ureteroscopy						
Flexible	5	13.95	0.007	71	0.88 (0.48–1.61)	0.68
Semirigid	3	2.53	0.28	21	0.70 (0.29–1.65)	0.41

OR odds ratio, RCT randomized controlled trials.

#### Publication bias

Base on the SFR, there was no evidence of significant publication bias from the Egger test (t = 0.77, P = 0.47). A well-proportioned funnel plot was formed in a sensitivity analysis using the trim-and-fill method. After the performance of trim-and-fill method, we observed the OR was 0.78 (95% CI = 0.49–1.23, P = 0.28), which indicated the result of trim-and-fill method didn’t convert comparatively.

### Secondary outcomes

#### Intraoperative complications

The intraoperative complications comprising bleeding, perforation and avulsion etc. Analyzing the data of 3 studies [11,12,14] with random effects model, including 2428 procedures, yielded no significant difference between case and control groups in terms of intraoperative complications (OR = 1.16, 95% CI 0.81–7.69, P = 0.88, Fig 2C) with a moderate heterogeneity among trials (Q = 6.53, P = 0.04, I^2^ = 69%, Fig 2C).

#### Postoperative complications

There were 6 articles [8,9,11,13–15] providing data with regard to postoperative complications such as bleeding, fever, urinary tract infection, bladder cramps, lung embolism, sepsis and so forth. However, two articles [9,11] with no events should be excluded because they didn’t provide any indication of the direction and magnitude of the relative treatment effect. Combining the results of studies with a fixed-effects model produced an OR of 1.46 (95% CI 1.06–2.00, P = 0.02; Q = 2.61, P = 0.45, I^2^ = 0%; Fig 2D), which comes into a conclusion that UAS group get a significant higher incidence of postoperative complications than non-UAS group.

#### Operative time

Six studies mentioned the operative time [8,9,11,13–15], whereas only 2 articles [13,14] provided the SD. Only half of articles with lacking data provided the value of range [9,11], which was essential to estimate SD with the theory of Hozo et al [19]. However, due to the limited sample size provided by De Sio et al [9], it was excluded from the meta-analysis on this endpoint. Finally, three studies [11,13,14] including 2514 procedures were obtained and showed no significant difference between arms (MD = 4.09, 95% CI -15.08–23.26, P = 0.68, Fig 2E) with a severe heterogeneity (Q = 71.48, P < 0.00001, I^2^ = 97%, Fig 2E).

#### Hospitalization duration

There were only two articles that compared the difference between arms regarding hospitalization duration [13,14]. Berquet et al [13] demonstrated no significant difference between groups concerning this issue. Whereas, Traxer et al indicated that hospitalization periods were longer in cases treated without a UAS [14]. Pooling the data of these articles, meta-analysis for hospitalization duration showed no significant difference between groups (MD = -0.13, 95% CI -0.32–0.06, P = 0.18; Q = 1.47, P = 0.22, I^2^ = 32%; Fig 2F).

## Discussion

Several articles had examined the effect of using UAS during ureteroscopy. However, the results of these studies were contradictory, even proposed completely different recommendations [8–15]. To the best of our knowledge, this is the first meta-analysis to elaborate the relationship between those contradictions, which have pointed out an evidential orientation on this issue. Besides, it should be noted that a series of subgroup analyses such as age, diseased region, study design, the control of stone size and the use of semirigid ureteroscope have been conducted. Only the subgroup of study design revealed the different result with quondam one, which indicated the stability of this meta-analysis. Moreover, both of Egger test and trim-and-fill method indicated no publication bias in this meta-analysis.

The efficacy and safety of UAS during ureteroscopy with a systematic review and meta-analysis was evaluated. In general, 8 studies containing 3099 patients, a total of 3127 procedures were included. The results indicated the same effect on SFR, intraoperative complications, operative time and hospitalization duration but showed a significant increase in the incidence of postoperative complications in UAS arm.

Stone burden was no significant differences in 6 original documents [8,10,11,13–15]. Subgroup analysis of the control of stone burden showed the same with original result, which indicated the reliability of outcome of SFR. Proponents of UAS proposed that the use of UAS could serve to remove stone fragments with basket easily. Also, it provided a repeatable, safe and fast access to the upper urinary tract with improved vision [5]. Therefore, the potential superiority of UAS in the SFR has been advocated. However, Berquet et al [13] documented the number of stone, stone location and the use of UAS had no effect on SFR. The only predictive factor of SFR was stone burden. Traxer et al [14] explored the effect of UAS with a sizable population of prospective study indicated that the SFR was 73.9% in UAS group versus 82.8% in non-UAS group, and presented that UAS is not used to increase SFR.

Some of the advantage of UAS have been recommended, such as providing an access to the collecting system with the ability for multiple entrie and exits of the ureter, decreasing intrarenal pressure during pulse or continuous ureteroscopic irrigation, and also improving drainage around the scope and visibility, thus protecting the scope when performing lithotripsy and extracting stone fragments [3–6]. However, several articles have indicated that UAS could be responsible for postoperative complications such as persistent hematuria, ureteral stricture, urinary extravasation etc [13,14,26]. In this meta-analysis, the higher postoperative complication was observed in UAS group. Ureteral injury with UAS insertion resulting in more postoperative persistent hematuria, use of ureteral stents, post-operative pain, and even contributing to ureteral strictures [26,27]. Lallas et al [28] observed that nadir ureteral blood flow was 25%, 70%, and 80% below baseline when 10/12F, 12/14F, and 14/16F UAS were used during ureteroscopy, while the bigger size of UAS need more time to recover. Traxer et al [7] indicated that severe injury of ureteral smooth muscle layers is common even after a 12/14Fr UAS was inserted. Other studies [5,29] also have shown that the insertion of the UAS could harm the ureteral mucosa and induce ischemia of the ureter. Furthermore, up-regulation of pro-inflammatory mediators, COX-2 and TNF-alpha were observed in the ureteral wall after the use of UAS, which might indicate another reason for postoperative pain and complications [30].

Among the studies for operative time, the synthesis of meta-analysis revealed the same effect between groups. Of note, with the estimation of SD from Pardalidis et al [11], it showed an inescapable deviation. Also, Kourambas et al [8] preferred the UAS group as they showed an advantage in operative time. On the other hand, Geraghty et al [15] and De Sio et al [9] demonstrated no significant difference between both arms. These results should not be neglected.

The following limitations were deserved mention in this meta-analysis. Firstly, this study was based on non-RCTs which meant the risk of bias from inappropriate random sequence generation and blinding was unavoidable. Secondly, several parameters indicated heterogeneity because of the discrepancy in study design, surgeon’s experience and some unmeasured confounders across studies. Finally, lack of the unified standard and the value of SD in some studies also prevented a more accurate analysis.

## Conclusions

To sum up, this is the first meta-analysis to evaluate the efficacy and safety of UAS during ureteroscopy and provides evidence that using a UAS in ureteroscopy neither has an effect on SFR, operative time, hospitalization time, nor intraoperative complications but significantly increases the incidence of postoperative complications. Current study didn’t manifest an obvious advantage for using a UAS during ureteroscopy, which indicated the use of UAS should not be a routine in all cases. However, given intrinsic restrictions of included studies, more RCTs are warranted to confirm and update the findings of this study.

## Supporting information

S1 Fig Egger test. (TIF)

S2 Fig Trim-and-fill method. (TIF)

S1 Table PRISMA 2009 checklist. (DOC)

S1 File The protocol from PROSPERO. (CRD42017052327).
(PDF)

S1 Text The search strategy for PubMed. (DOCX)
