# Supplementary material for: Use of the ureteral access sheath during ureteroscopy: A systematic review and meta-analysis
Source: PLoS One. 2018 Feb 28;13(2):e0193600. doi: 10.1371/journal.pone.0193600 (PMC5831629; doi:10.1371/journal.pone.0193600)
Supplement: S1 File — (CRD42017052327). (PDF) [file pone.0193600.s004.pdf]

Systematic review

To edit the record click Start an update below. This will create a new version of the record - the existing version will remain unchanged.

1. * Review title.

Give the working title of the review, for example the one used for obtaining funding. Ideally the title should state succinctly the interventions or exposures being reviewed and the associated health or social problems. Where appropriate, the title should use the PI(E)COS structure to contain information on the Participants, Intervention (or Exposure) and Comparison groups, the Outcomes to be measured and Study designs to be included.

The effect of ureteroscopy with and without the use of ureteral access sheaths: a systematic review and meta-analysis

2. Original language title.

For reviews in languages other than English, this field should be used to enter the title in the language of the review. This will be displayed together with the English language title.

3. * Anticipated or actual start date.

Give the date when the systematic review commenced, or is expected to commence.

14/11/2016

4. * Anticipated completion date.

Give the date by which the review is expected to be completed.

31/08/2018

5. * Stage of review at time of this submission.

Indicate the stage of progress of the review by ticking the relevant Started and Completed boxes. Additional information may be added in the free text box provided.

Please note: Reviews that have progressed beyond the point of completing data extraction at the time of initial registration are not eligible for inclusion in PROSPERO. Should evidence of incorrect status and/or completion date being supplied at the time of submission come to light, the content of the PROSPERO record will be removed leaving only the title and named contact details and a statement that inaccuracies in the stage of the review date had been identified.

This field should be updated when any amendments are made to a published record and on completion and publication of the review.

The review has not yet started: No

Review stage	Started	Completed
Preliminary searches	Yes	No
Piloting of the study selection process	No	No
Formal screening of search results against eligibility criteria	No	No
Data extraction	No	No

Review stage	Started	Completed
Risk of bias (quality) assessment	No	No
Data analysis	No	No

Provide any other relevant information about the stage of the review here (e.g. Funded proposal, protocol not yet finalised).

Protocol not yet finalised.
Protocol not yet finalised.

6. * Named contact.

The named contact acts as the guarantor for the accuracy of the information presented in the register record.

Jian Huang

Email salutation (e.g. "Dr Smith" or "Joanne") for correspondence:

7. * Named contact email.

Give the electronic mail address of the named contact.

huangjianmnwk@163.com

8. Named contact address

PLEASE NOTE this information will be published in the PROSPERO record so please do not enter private information

Give the full postal address for the named contact.

The First Affiliated Hospital of Guangzhou Medical University, Kangda Road, Haizhu District, Guangzhou City, Guangdong Province, China

9. Named contact phone number.

Give the telephone number for the named contact, including international dialling code.

15889830986

10. * Organisational affiliation of the review.

Full title of the organisational affiliations for this review and website address if available. This field may be completed as 'None' if the review is not affiliated to any organisation.

The First Affiliated Hospital of Guangzhou Medical University

Organisation web address:
http://www.gzhmc.edu.cn/

11. Review team members and their organisational affiliations.

Give the title, first name, last name and the organisational affiliations of each member of the review team. Affiliation refers to groups or organisations to which review team members belong.

Mr Jian Huang. First Affiliated Hospital of Guangzhou Medical University
Mr Zhijian Zhao. First Affiliated Hospital of Guangzhou Medical University
Mr Xiongfa Liang. First Affiliated Hospital of Guangzhou Medical University
Dr Tao Zeng. First Affiliated Hospital of Guangzhou Medical University
Dr Luhao Liu. First Affiliated Hospital of Guangzhou Medical University
[illegible]

Professor Wenqi Wu. First Affiliated Hospital of Guangzhou Medical University

12. * Funding sources/sponsors.

Give details of the individuals, organizations, groups or other legal entities who take responsibility for initiating, managing, sponsoring and/or financing the review. Include any unique identification numbers assigned to the review by the individuals or bodies listed.

Department of Urology, First Affiliated Hospital of Guangzhou Medical University; Funding provided by Wenqi Wu

13. * Conflicts of interest.

List any conditions that could lead to actual or perceived undue influence on judgements concerning the main topic investigated in the review.

None

14. Collaborators.

Give the name and affiliation of any individuals or organisations who are working on the review but who are not listed as review team members.

15. * Review question.

State the question(s) to be addressed by the review, clearly and precisely. Review questions may be specific or broad. It may be appropriate to break very broad questions down into a series of related more specific questions. Questions may be framed or refined using PI(E)COS where relevant.

Does a ureteral access sheath improve the stone-free rates in patients undergoing ureteroscopy?

How does the use of a ureteral access sheath impact on hospital stay after ureteroscopy?

How does a ureteral access sheath impact on mean operative time in patients undergoing ureteroscopy?

Does the use of a ureteral access sheath improve the incidence of complications after ureteroscopy?

16. * Searches.

Give details of the sources to be searched, search dates (from and to), and any restrictions (e.g. language or publication period). The full search strategy is not required, but may be supplied as a link or attachment.

We will search the following electronic bibliographic databases: PubMed, EMBASE, MEDLINE, Web of Science and the Cochrane Library.

The search strategy will include only terms relating to or describing the intervention. The terms will be combined with the Countway Library of Medicine filter for controlled trials of interventions. The search strategy for MEDLINE will be made available in the published protocol. Search terms will be adapted for use with other bibliographic databases in combination with database-specific filters for controlled trials, where these are available.

There will be no language restrictions. Studies published between January 1990 and the date the searches are run will be sought. The searches will be re-run just before the final analyses and further studies retrieved for inclusion.

17. URL to search strategy.

Give a link to the search strategy or an example of a search strategy for a specific database if available (including the keywords that will be used in the search strategies).

Yes I give permission for this file to be made publicly available

18. * Condition or domain being studied.

Give a short description of the disease, condition or healthcare domain being studied. This could include health and wellbeing outcomes.

The ureteral access sheath (UAS) has been developed using the same concept as the AMPLATZ sheath for percutaneous nephrolithotomy, i.e., to allow direct access to the kidney and to decrease the intra-renal pressure during upper-tract endourological procedures.

19. * Participants/population.

Give summary criteria for the participants or populations being studied by the review. The preferred format includes details of both inclusion and exclusion criteria.

Inclusion: Without the combination of other therapy.

Exclusion: Absence of key information such as sample size, hazard ratio (HR) and risk ratio (RR), 95% CI, and P value.

20. * Intervention(s), exposure(s).

Give full and clear descriptions or definitions of the nature of the interventions or the exposures to be reviewed.

The ureteral access sheath (UAS) has been developed using the same concept as the AMPLATZ sheath for percutaneous nephrolithotomy, i.e., to allow direct access to the kidney and to decrease the intra-renal pressure during upper-tract endourological procedures. It has become increasingly popular as it offers a number of potential advantages including facilitation of access to renal collecting systems, multiple entry and re-entry, decreased intra-renal pressure, and improved drainage around the scope.

21. * Comparator(s)/control.

Where relevant, give details of the alternatives against which the main subject/topic of the review will be compared (e.g. another intervention or a non-exposed control group). The preferred format includes details of both inclusion and exclusion criteria.

Ureteroscopy without the use of a ureteral access sheath.

22. * Types of study to be included.

Give details of the types of study (study designs) eligible for inclusion in the review. If there are no restrictions on the types of study design eligible for inclusion, or certain study types are excluded, this should be stated. The preferred format includes details of both inclusion and exclusion criteria.

We will include randomised trials to assess the beneficial effects of the treatments, and will supplement these with observational studies (including cohort and case-control studies) for the assessment of harms.

23. Context.

Give summary details of the setting and other relevant characteristics which help define the inclusion or exclusion criteria.

This review is being undertaken to compare the effect of ureteroscopy with and without the use of ureteral access sheaths.

24. * Primary outcome(s).

Give the pre-specified primary (most important) outcomes of the review, including details of how the outcome is defined and measured and when these measurement are made, if these are part of the review inclusion criteria.

Stone-free rates.

Timing and effect measures

Stone-free status to be evaluated 3 months after the surgery using abdominal ultrasound or a CT scan. Based on most publications, it is considered that the patient is stone-free if there is less than one residual fragment left with a diameter <3 mm.

25. * Secondary outcome(s).

List the pre-specified secondary (additional) outcomes of the review, with a similar level of detail to that required for primary outcomes. Where there are no secondary outcomes please state 'None' or 'Not applicable' as appropriate to the review

Hospital stay, operative time and the incidence of complications.

Timing and effect measures

Operating time is defined as the time from the insertion of the endoscope until the insertion of a bladder catheter.

26. Data extraction (selection and coding).

Give the procedure for selecting studies for the review and extracting data, including the number of researchers involved and how discrepancies will be resolved. List the data to be extracted.

Two investigators will search the publications independently. If the two investigators achieve different results, an independent expert in urology will make the final decision regarding inclusion. Information collected from these publications will include: first author, year of publication, stone burden, number of patients, patient characteristics, study design (blinded or not), and outcomes. The primary outcome is stone-free rate. Others outcomes include: hospital stay, operative time and complications.

27. * Risk of bias (quality) assessment.

State whether and how risk of bias will be assessed (including the number of researchers involved and how discrepancies will be resolved), how the quality of individual studies will be assessed, and whether and how this will influence the planned synthesis.

Two review authors will independently assess the risk of bias in included studies by considering the following characteristics:

Randomisation sequence generation: was the allocation sequence adequately generated?

Treatment allocation concealment: was the allocated treatment adequately concealed from study participants and clinicians and other healthcare or research staff at the enrolment stage?

Blinding: were the personnel assessing outcomes and analysing data sufficiently blinded to the intervention allocation throughout the trial?

Completeness of outcome data: were participant exclusions, attrition and incomplete outcome data adequately addressed in the published report?

Selective outcome reporting: is there evidence of selective outcome reporting and might this have affected the study results?

Other sources of bias: was the trial apparently free of any other problems that could produce a high risk of bias?

28. * Strategy for data synthesis.

Give the planned general approach to synthesis, e.g. whether aggregate or individual participant data will be used and whether a quantitative or narrative (descriptive) synthesis is planned. It is acceptable to state that a quantitative synthesis will be used if the included studies are sufficiently homogenous.

We will provide a narrative synthesis of the findings from the included studies, structured around the type of intervention, the target population characteristics, the type of outcome and the intervention content. We will provide summaries of intervention effects for each study by calculating risk ratios (for dichotomous outcomes) or standardised mean differences (for continuous outcomes).

We anticipate that there will be limited scope for meta-analysis because of the range of different outcomes measured across the small number of existing trials. However, where studies have used the same type of intervention and comparator, with the same outcome measure, we will pool the results using a random-effects meta-analysis, with standardised mean differences for continuous outcomes and risk ratios for binary outcomes, and will calculate 95% confidence intervals and two sided P values for each outcome. In studies where the effects of clustering have not been taken into account, we will adjust the standard deviations for the design effect. Heterogeneity between the studies in effect measures will be assessed using both the chi-square test and the I-squared statistic. We will consider an I-squared value greater than 50% as beinig

indicative of substantial heterogeneity. We will conduct sensitivity analyses based on study quality, and we will use stratified meta-analyses to explore heterogeneity in effect estimates according to: study quality; study populations; the logistics of the intervention provision; and intervention content. We will also assess evidence of publication bias.

29. * Analysis of subgroups or subsets.

Give details of any plans for the separate presentation, exploration or analysis of different types of participants (e.g. by age, disease status, ethnicity, socioeconomic status, presence or absence or co-morbidities); different types of intervention (e.g. drug dose, presence or absence of particular components of intervention); different settings (e.g. country, acute or primary care sector, professional or family care); or different types of study (e.g. randomised or non-randomised).

If the necessary data are available, subgroup analyses will be done for randomised or non-randomised clinical trials. We also plan to perform a subgroup analysis by preoperative stent use and non-use.

30. * Type and method of review.

Select the type of review and the review method from the lists below. Select the health area(s) of interest for your review.

Type of review

Cost effectiveness	No
Diagnostic	No
Epidemiologic	No
Individual patient data (IPD) meta-analysis	No
Intervention	No
Meta-analysis	Yes
Methodology	No
Network meta-analysis	No
Pre-clinical	No
Prevention	No
Prognostic	No
Prospective meta-analysis (PMA)	No
Qualitative synthesis	No
Review of reviews	No
Service delivery	No
Systematic review	Yes
Other	No

Health area of the review

Alcohol/substance misuse/abuse	No
Blood and immune system	No

Cancer	No
Cardiovascular	No
Care of the elderly	No
Child health	No
Complementary therapies	No
Crime and justice	No
Dental	No
Digestive system	No
Ear, nose and throat	No
Education	No
Endocrine and metabolic disorders	No
Eye disorders	No
General interest	No
Genetics	No
Health inequalities/health equity	No
Infections and infestations	No
International development	No
Mental health and behavioural conditions	No
Musculoskeletal	No
Neurological	No
Nursing	No
Obstetrics and gynaecology	No
Oral health	No
Palliative care	No
Perioperative care	No
Physiotherapy	No
Pregnancy and childbirth	No
Public health (including social determinants of health)	No
Rehabilitation	No

Respiratory disorders	No
Service delivery	No
Skin disorders	No
Social care	No
Surgery	No
Tropical Medicine	No
Urological	No
Wounds, injuries and accidents	No
Violence and abuse	No

31. Language.

Select each language individually to add it to the list below, use the bin icon to remove any added in error.

English

There is an English language summary.

32. Country.

Select the country in which the review is being carried out from the drop down list. For multi-national collaborations select all the countries involved.

China

33. Other registration details.

Give the name of any organisation where the systematic review title or protocol is registered (such as with The Campbell Collaboration, or The Joanna Briggs Institute) together with any unique identification number assigned. (N.B. Registration details for Cochrane protocols will be automatically entered). If extracted data will be stored and made available through a repository such as the Systematic Review Data Repository (SRDR), details and a link should be included here. If none, leave blank.

34. Reference and/or URL for published protocol.

Give the citation and link for the published protocol, if there is one

Yes I give permission for this file to be made publicly available

35. Dissemination plans.

Give brief details of plans for communicating essential messages from the review to the appropriate audiences.

Do you intend to publish the review on completion?

Yes

36. Keywords.

Give words or phrases that best describe the review. Separate keywords with a semicolon or new line. Keywords will help users find the review in the Register (the words do not appear in the public record but are included in searches). Be as specific and precise as possible. Avoid acronyms and abbreviations unless these are in wide use.

systematic review

meta-analysis

ureteral access sheath

ureteroscopy

37. Details of any existing review of the same topic by the same authors.

Give details of earlier versions of the systematic review if an update of an existing review is being registered, including full bibliographic reference if possible.

38. * Current review status.

Review status should be updated when the review is completed and when it is published.

Review_Ongoing

39. Any additional information.

Provide any other information the review team feel is relevant to the registration of the review.

40. Details of final report/publication(s).

This field should be left empty until details of the completed review are available.
