# Supplementary material for: Use of the ureteral access sheath during ureteroscopy: A systematic review and meta-analysis
Source: PLoS One. 2018 Feb 28;13(2):e0193600. doi: 10.1371/journal.pone.0193600 (PMC5831629; doi:10.1371/journal.pone.0193600)
Supplement: S1 Text — (DOCX) [file pone.0193600.s005.docx]

**The search strategy for PubMed**

(("randomized controlled trial"[pt] OR "controlled clinical trial"[pt] OR "clinical trials as topic"[mesh] OR "random allocation"[mesh] OR "double-blind method"[mesh] OR "single-blind method"[mesh] OR "clinical trial"[pt] OR "research design"[mesh:noexp] OR "comparative study"[pt] OR "evaluation studies"[pt] OR "follow-up studies"[mesh] OR "prospective studies"[mesh] OR "cross-over studies"[mesh] OR "clinical trial"[tw] OR ((singl*[tw] OR doubl*[tw] OR trebl*[tw]) AND (mask*[tw] OR blind*[tw])) OR placebo*[tw] OR random*[tw] OR "control"[tw] OR "controls"[tw] OR prospecitv*[tw] OR volunteer*[tw])) AND (((((((FURS[Title/Abstract]) OR Ureteroscopic lithotripsy[Title/Abstract]) OR retrograde intrarenal surgery[Title/Abstract]) OR (((((((((((((RIRS[Title/Abstract]) OR URL[Title/Abstract]) OR URS[Title/Abstract]) OR ((((((((flexible ureteroscope[Title/Abstract]) OR flexible ureteroscopy[Title/Abstract]) OR semirigid ureteroscope[Title/Abstract]) OR semirigid ureteroscopy[Title/Abstract]) OR ureteroscope[Title/Abstract]) OR ureteroscopy[Title/Abstract]) OR ureteroscopic[Title/Abstract]) OR Ureterolithotripsy[Title/Abstract]))) OR "Ureteroscopy"[Mesh])) OR "Ureteroscopes"[Mesh])))))))) AND ((((((((((((((sheath[Title/Abstract]) OR sheaths[Title/Abstract]) OR ureter sheath[Title/Abstract]) OR ureteral access sheath[Title/Abstract]) OR ureteral access sheaths[Title/Abstract]) OR UAS[Title/Abstract]) OR Access sheath[Title/Abstract]) OR Access sheaths[Title/Abstract]) OR ureter sheaths[Title/Abstract])))))))
